# Detection and identification of fungi in the lower airway of children with and without cystic fibrosis

**DOI:** 10.3389/fmicb.2023.1119703

**Published:** 2023-02-09

**Authors:** John B. O’Connor, Brandie D. Wagner, J. Kirk Harris, Daniel N. Frank, Diana E. Clabots, Theresa A. Laguna

**Affiliations:** ^1^Division of Pulmonary and Sleep Medicine, Ann & Robert H. Lurie Children’s Hospital of Chicago, Chicago, IL, United States; ^2^University of Colorado School of Medicine, Aurora, CO, United States; ^3^Colorado School of Public Health, University of Colorado Denver, Aurora, CO, United States; ^4^Department of Internal Medicine, Palmetto General Hospital, Hialeah, FL, United States; ^5^Department of Pediatrics, Northwestern University Feinberg School of Medicine, Chicago, IL, United States

**Keywords:** cystic fibrosis, mycobiome, pediatrics, SSU-rRNA sequencing, bronchoalveolar lavage, infection, inflammation

## Abstract

**Introduction:**

Airway infection and inflammation lead to the progression of obstructive lung disease in persons with cystic fibrosis (PWCF). However, cystic fibrosis (CF) fungal communities, known drivers of CF pathophysiology, remain poorly understood due to the shortcomings of traditional fungal culture. Our objective was to apply a novel small subunit rRNA gene (SSU-rRNA) sequencing approach to characterize the lower airway mycobiome in children with and without CF.

**Methods:**

Bronchoalveolar lavage fluid (BALF) samples and relevant clinical data were collected from pediatric PWCF and disease control (DC) subjects. Total fungal load (TFL) was measured using quantitative PCR, and SSU-rRNA sequencing was used for mycobiome characterization. Results were compared across groups, and Morisita-Horn clustering was performed.

**Results:**

161 (84%) of the BALF samples collected had sufficient load for SSU-rRNA sequencing, with amplification being more common in PWCF. BALF from PWCF had increased TFL and increased neutrophilic inflammation compared to DC subjects. PWCF exhibited increased abundance of *Aspergillus* and *Candida*, while *Malassezia*, *Cladosporium*, and Pleosporales were prevalent in both groups. CF and DC samples showed no clear differences in clustering when compared to each other or to negative controls. SSU-rRNA sequencing was used to profile the mycobiome in pediatric PWCF and DC subjects. Notable differences were observed between the groups, including the abundance of *Aspergillus* and *Candida*.

**Discussion:**

Fungal DNA detected in the airway could represent a combination of pathogenic fungi and environmental exposure (e.g., dust) to fungus indicative of a common background signature. Next steps will require comparisons to airway bacterial communities.

## Introduction

Chronic airway infection and inflammation leading to progressive, obstructive lung disease is the primary cause of morbidity and mortality in cystic fibrosis (CF; Gibson et al., 2003; LiPuma, 2015). Investigating the contribution of airway infection to the development and progression of CF lung disease in pediatric populations can further our understanding of how lung disease develops and inform therapeutic approaches. While traditional bacterial pathogens, including *Pseudomonas aeruginosa*, *Staphylococcus aureus*, *Haemophilus influenzae*, *Burkholderia cepacia complex, Stenotrophomonas maltophilia*, and *Achromobacter xylosoxidans* were thought to be the main drivers of airway damage and lung function decline in CF (Gibson et al., 2003; Burns and Rolain, 2014; LiPuma, 2015), culture-independent, next generation sequencing (NGS) approaches have sharpened our perspective of the CF airway to include more complex and dynamic polymicrobial bacterial communities (Rogers et al., 2004; Sibley et al., 2006; LiPuma, 2015; Tracy et al., 2015; O’Connor et al., 2021). Given the complex ecology in the lower CF airway, defining the contributions of non-bacterial pathogens, particularly fungi, to the development and progression of CF lung disease has now emerged as a priority (Blanchard and Waters, 2019).

Fungi have been detected by culture in airway secretions of PWCF and may be correlated with worse clinical outcomes (Amin et al., 2010; Saunders et al., 2016), including an increased risk of hospitalization and the progression of structural lung disease (Amin et al., 2010; Breuer et al., 2020). Additionally, fungi specifically found in the CF airway, including *Aspergillus*, *Scedosporium*, and *Candida*, have been associated with worsening lung function (Saunders et al., 2016; Al Shakirchi et al., 2020; Breuer et al., 2020). While the impact of fungi on lung health has been observed in CF, accurately detecting and identifying fungi by culture of airway secretions are limited by the lack of a standardized techniques across clinical laboratories, slow growth rates, and difficulty in identifying fungi (Borman et al., 2010; Chen et al., 2018). The development of an accurate NGS approach to detect and identify lower airway fungal communities, or the mycobiome, across the pediatric age spectrum, as has been previously done with bacterial communities (Rogers et al., 2004; Harris et al., 2007; Tracy et al., 2015; Zemanick et al., 2017; O’Connor et al., 2021), is crucial to inform our future understanding of the clinical impact of fungi in the lower airways of PWCF.

Our objective was to apply a novel, molecular sequencing-based approach to characterize the mycobiome in the ideal lower airway sample, BALF, which more accurately reflects the ecology of airway samples (Kirst et al., 2019) and is available from children who often cannot produce sputum. The ability to characterize the composition of the mycobiome in the lower airway of pediatric PWCF is the first step toward understanding the complex mechanisms driving infection and inflammation in CF lung disease.

## Materials and methods

### Study design and population

Bronchoalveolar lavage fluid was originally collected from PWCF and disease control (DC) subjects undergoing clinically indicated flexible bronchoscopy. The DC population consisted of children without a confirmed diagnosis of CF. BALF samples were collected under IRB-approved protocols at the University of Minnesota and Children’s Hospital of Colorado. Informed consent, adolescent assent, and parental permission along with Health Insurance Portability and Accountability Act (HIPPA) of 1996 authorization were obtained according to each institution’s IRB rules and regulations. Demographic and clinical data associated with the day of BALF sample collection, including past medical history, indication for bronchoscopy, lung function measurements, culture results, comorbidities, and cytology data were collected from the electronic medical record and the Cystic Fibrosis Foundation Patient Registry.

### Sample collection and processing

Flexible bronchoscopy was performed on children in accordance with each institution’s standard-of-care clinical guidelines. Excess BALF that was not needed for clinical testing was stored for future research. A portion of neat unprocessed sample was set aside. If there was sufficient sample volume remaining for processing, another portion was centrifuged at 250 × *g* for 10 min at 4°C. Following centrifugation, the pellet and supernatant were separated. Neat, pellet, and supernatant were then all stored at −80°C until analysis. Neat and pellet samples were transported overnight on dry ice to Children’s Hospital of Colorado for DNA extraction, quantitative PCR (qPCR) and SSU-rRNA gene sequencing. Both neat and pellet samples were included in the sequencing analysis as has been previously described (Harris et al., 2007; O’Connor et al., 2021).

### Mycobiome analysis

Total fungal load (TFL) was measured using a published TaqMan qPCR assay (Liu et al., 2012), which targets approximately 350 nucleotides in the V6–V8 variable region of the SSU-rRNA. The nucleotide region corresponds to *Saccharomyces cerevisiae* positions 1,199–1,549 (*Escherichia coli* positions 974–1,295). The assay was based on parameters that were published using a cloned fungal fragment (*S. cerevisiae*) for standards (10^3^–10^8^ copies, 10-fold dilution series, as standards). TFL was quantified as the logarithmic transformation of the copies per reaction. For fungal profiling, we utilized TFL to adjust template amount (up to maximum volume) when attempting PCR. While all samples were attempted, any with no apparent band after PCR were deemed to have insuffient load for amplification. DNA sequencing was performed using primers based on the fungal load qPCR assay (PCR primer sequences provided in Supplementary Figure 1) and utilizing a modified PCR approach to enable amplification from a larger range of fungal load (Williamson et al., 2021). PCR was performed in 100 μl total reaction volume split across four wells of a 96-well plate. This provides three independent amplification reactions with the sample DNA and an index-specific negative control to monitor bench assembly of PCR. The independent reactions containing template are meant to average initial rounds of amplification to mitigate PCR drift (Wagner et al., 1994), and are reconstituted into a single well (75 μl) amplicon sample for confirmation of amplification and subsequent sequencing steps. One of the sequencing primers was shortened relative to the qPCR assay to generate a 3′ mismatch between the primer and the human SSU-rRNA gene to improve the ratio of fungal DNA amplification. Analyses were performed on negative control samples, which consisted of only assay reagents, to assess background as has been done previously (O’Connor et al., 2021; Williamson et al., 2021).

### Analysis of illumina paired-end reads

Illumina MiSeq paired-end reads were aligned to human reference genome hg19 with bowtie2 and matching sequences discarded (iGenomes^1^; Langmead and Salzberg, 2012). As previously described, the remaining non-human paired-end sequences were sorted by sample *via* barcodes in the paired reads with a python script (Markle et al., 2013). The data presented in the study are deposited the NCBI Sequence Read Archive, accession number PRJNA922524 (https://www.ncbi.nlm.nih.gov/bioproject/PRJNA922524). The sorted paired reads were assembled using phrap (Ewing et al., 1998; Ewing and Green, 1998). Pairs that did not assemble were discarded. Assembled sequence ends were trimmed over a moving window of five nucleotides until average quality met or exceeded 20. Trimmed sequences with more than one ambiguity or shorter than 250 nt were discarded. Assembled sequences were aligned and classified with SINA (1.3.0-r23838) (Pruesse et al., 2012) using the 10,265 fungal sequences in Silva 138.1NR99 (Quast et al., 2012) as reference configured to yield the Silva taxonomy. Operational taxonomic units (OTUs) were produced by clustering sequences with identical taxonomic assignments. The FungiFQV2 primers generated 9,802,508 sequences for 168 samples (average sequence length: 315 nt; average sample size: 56,771 sequences/sample; minimum sample size: 5,282; and maximum sample size: 583,650). The median Goods coverage score was ≥ 99.65% at the rarefaction point of 5,282 sequences. The software package Explicet (v2.10.5, www.explicet.org; Robertson et al., 2013) was used for display, analysis (rarefied values for alpha diversity metrics).

### Data processing and statistical analysis

Persons with cystic fibrosis (PWCF) and DC subjects were compared using Wilcoxon rank sum tests for continuous variables and chi-square or Fisher’s Exact test for categorical variables. To account for differences in sequencing depth, the relative abundance (RA) of each taxon was calculated (100*number of sequences for specific taxon/total number of sequences). The Shannon diversity index was calculated to measure the alpha diversity. Cubic B splines were used to model the relationships between microbial variables, including TFL and diversity, and age by disease group as has been previously described (O’Connor et al., 2021). Spearman correlations were used to assess the correlations between continuous variables. The Morisita-Horn beta diversity measure was used as a distance metric of samples sorted from the same 18S SSU-rRNA mycobiome data set for both Principal Coordinate Analysis (PCoA) and unsupervised hierarchical clustering analysis (HCA) with complete linkage.

## Results

### Sample population

One hundred ninety-one samples were collected from 128 (67%) DCs and 63 (33%) PWCF. Patient demographics are summarized in Table 1. CF subjects were significantly older at the time of BALF sample collection and had significantly increased cellular markers of inflammation. Indications for flexible bronchoscopy and primary medical diagnoses for the DC group were previously reported (O’Connor et al., 2021).

**Table 1:** Data are presented as *n*, median (range) or *n* (%), unless otherwise stated.

	DCs (*n* = 128)	CF (*n* = 63)	*p* Value
Age years, median (range)	5.2 (0–21.0)	9.7 (0.9–19.7)	<0.001
<2 years, number (%)	38 (30%)	3 (5%)	<0.001
2–5 years, number (%)	31 (24%)	9 (14%)	
6–10 years, number (%)	32 (25%)	25 (40%)	
11–17 years, number (%)	17 (13%)	21 (33%)	
18 years and older, number (%)	10 (8%)	5 (8%)	
Female, number (%)	60 (47%)	41 (65%)	0.018
Weight (kg), median (range; data available)	18.2 (4.2–78.3; *N* = 128)	29.0 (13.5–74.7; *N* = 49)	<0.001
Height (cm), median (range; data available)	106.0 (51.0–181.0; *N* = 120)	132.4 (97.8–175.4; *N* = 49)	<0.001
Genotype, data available	N/A	*N* = 62	N/A
F508del/F508del, number (%)	N/A	36 (58%)	N/A
F508del/other, number (%)	N/A	22 (35%)	N/A
Other/other, number (%)	N/A	4 (6%)	N/A
FEV1% predicted, median (range; data available)	83.0 (41.0–109.0; *N* = 27)	86.5 (35.0–131.0; *N* = 50)	0.709
BALF cell counts, data available	*N* = 126	*N* = 53	N/A
White blood cells, median (range; data available)	213 (0–8,122; *N* = 126)	1,312 (66–51,500; *N* = 53)	<0.001
Percent neutrophils, median (range; data available)	8 (1–99; *N* = 107)	80 (3–99; *N* = 51)	<0.001
Percent lymphocytes, median (range; data available)	9 (1–66; *N* = 155)	3 (0–28; *N* = 39)	<0.001
Positive bacterial culture, number (% of those with data available;number with data available)	**43 (34%**; ***N*** = **126)**	**28 (48%**; ***N* = 58)**	0.067
Fungal culture performed	6 (5%)	22 (35%)	<0.001
Positive fungal culture, number (% of those with data available)	**4 (67%)**	**6 (27%)**	0.417
Antibiotic use number (% of those with data available; number with data available)	35 (90%; *N* = 39)	28 (45%; *N* = 62)	<0.001^*^

CF, cystic fibrosis; FEV1, forced expiratory volume in 1 s; BALF, bronchoalveolar lavage fluid; ^*^p value calculated using Fisher’s exact test.

### Fungal load

**Figure 1:**
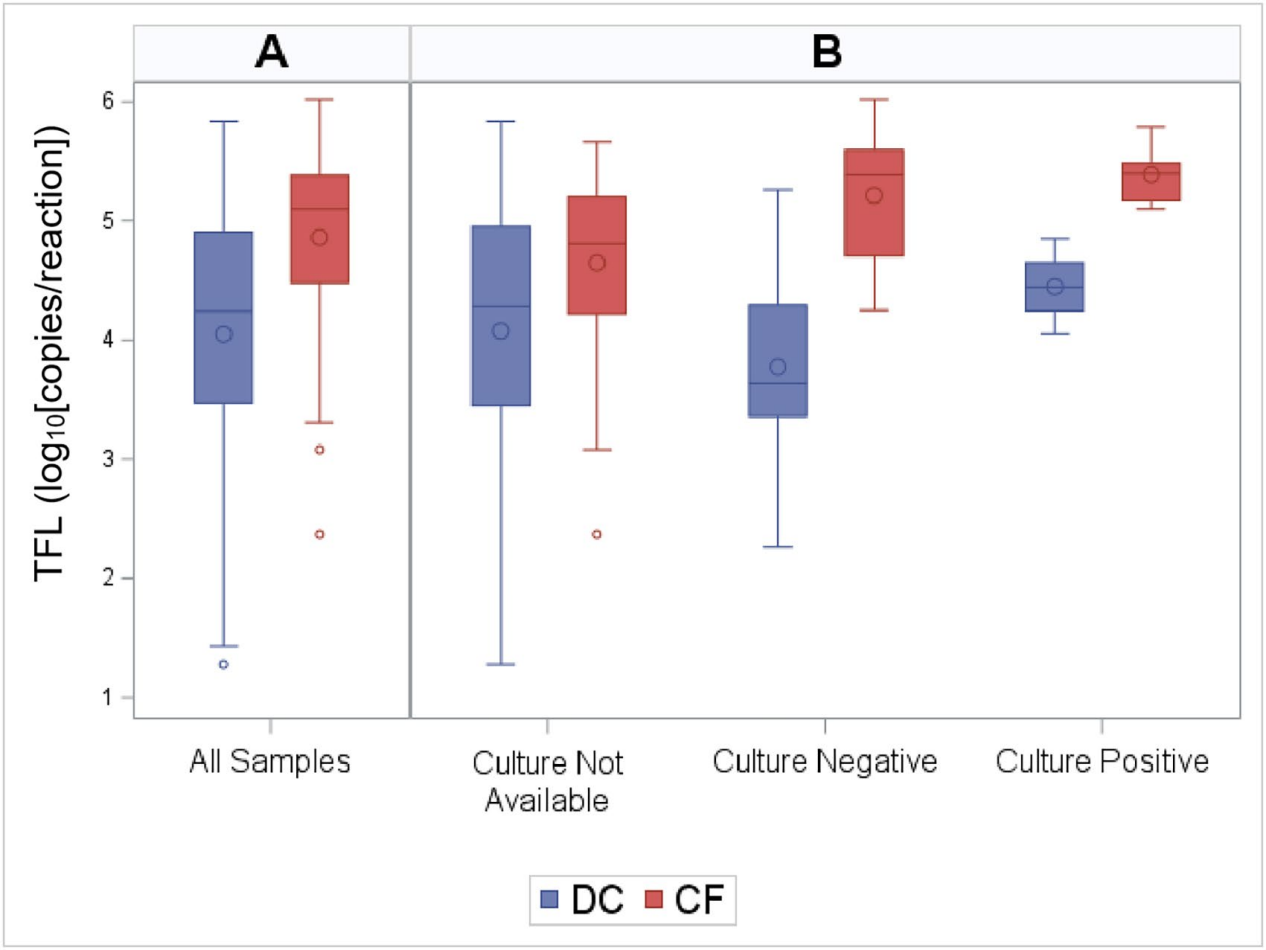
Total fungal load (TFL) by CF status and culture results. Distribution of TFL grouped by **(A)** CF status alone, and **(B)** CF status along with culture results. The box indicates the interquartile range (IQR; 25—75th percentile), and the median and mean are indicated by the lines and large circles, respectively. Whiskers indicate data within 1.5 times the IQR, and points indicate individual data values. TFL as measured by qPCR was higher in PWCF when compared to DC subjects (Wilcoxon, *p* < 0.01).

**Figure 2:**
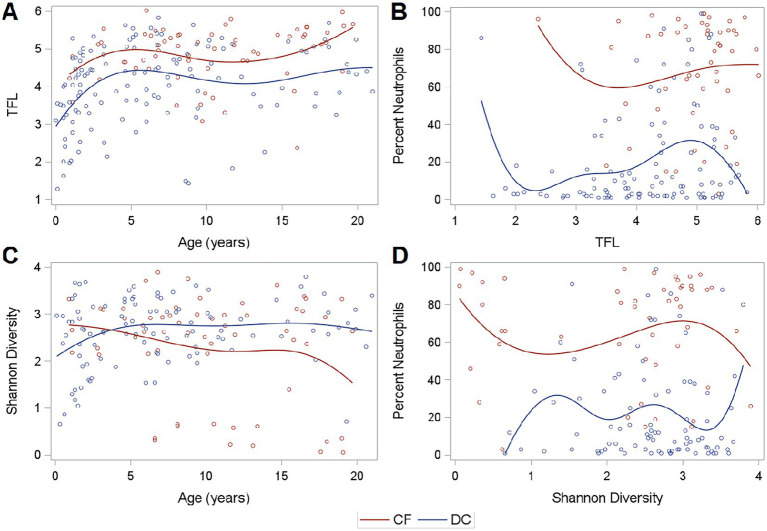
Fungal load, diversity, and neutrophilic inflammation. **(A)** TFL across age, **(B)** Neutrophilic Inflammation across TFL, **(C)** Shannon Diversity across age, and **(D)** Neutrophilic Inflammation across Shannon Diversity. Curves are generated using a cubic B spline scatterplot smoother.

Total fungal load as measured by qPCR was higher in PWCF when compared to DC subjects (Wilcoxon, *p* < 0.01; Figure 1A). This was true across the age spectrum (Figure 2A), with increased fungal burden observed with increased age at collection in both CF (Spearman Coefficient = 0.25, *p* = 0.05) and DC (Spearman Coefficient = 0.32, *p* < 0.01) populations. Median TFL was slightly higher in samples with positive fungal cultures (Figure 1B), though it did not show statistical significance [median (IQR): 5.14 (4.55–5.41) in culture positive compared to 4.48 (3.64–5.38) in culture negative, Wilcoxon, *p* = 0.24]. Culture-negative samples exhibited a wider interquartile range (IQR) of TFL values compared to culture-positive samples. Additionally, a wider and lower IQR was observed for samples with no culture data available (Figure 1B). Background TFL was detected in very low amounts by qPCR, with an average of only 0.9 log10 copies per reaction in negative controls.

### Sequencing

Of the 191 samples, 161 (84%) had sufficient load for sequencing, meaning they had an apparent band after PCR. TFL was higher in samples that were successfully sequenced [median (IQR): 4.70 (4.05–5.23) in sequenced samples compared to 2.57 (2.06–3.10) in non-sequenced samples, Wilcoxon, *p* < 0.01]. Patient demographics for samples that were successfully sequenced are summarized in Supplementary Table 1. CF samples exhibited increased frequency of sufficient amplification (Chi-squared, *p* < 0.01) with 60 (95%) being successfully sequenced.

### Airway composition and diversity

Of the successfully sequenced samples, the median (IQR) Shannon alpha diversity measures were 2.64 (2.15–2.08) for CF samples and 2.68 (2.23–3.10) for DC Samples indicating no difference in mycobiome diversity (Wilcoxon, *p* = 0.29). However, PWCF exhibited slightly decreased diversity at older ages, though these findings were driven by a subset of individuals (Figure 2C). Shannon diversity was not associated with neutrophilic inflammation (Figure 2D). The relationship between Shannon diversity and TFL is shown in Supplementary Figure 2.

**Figure 3:**
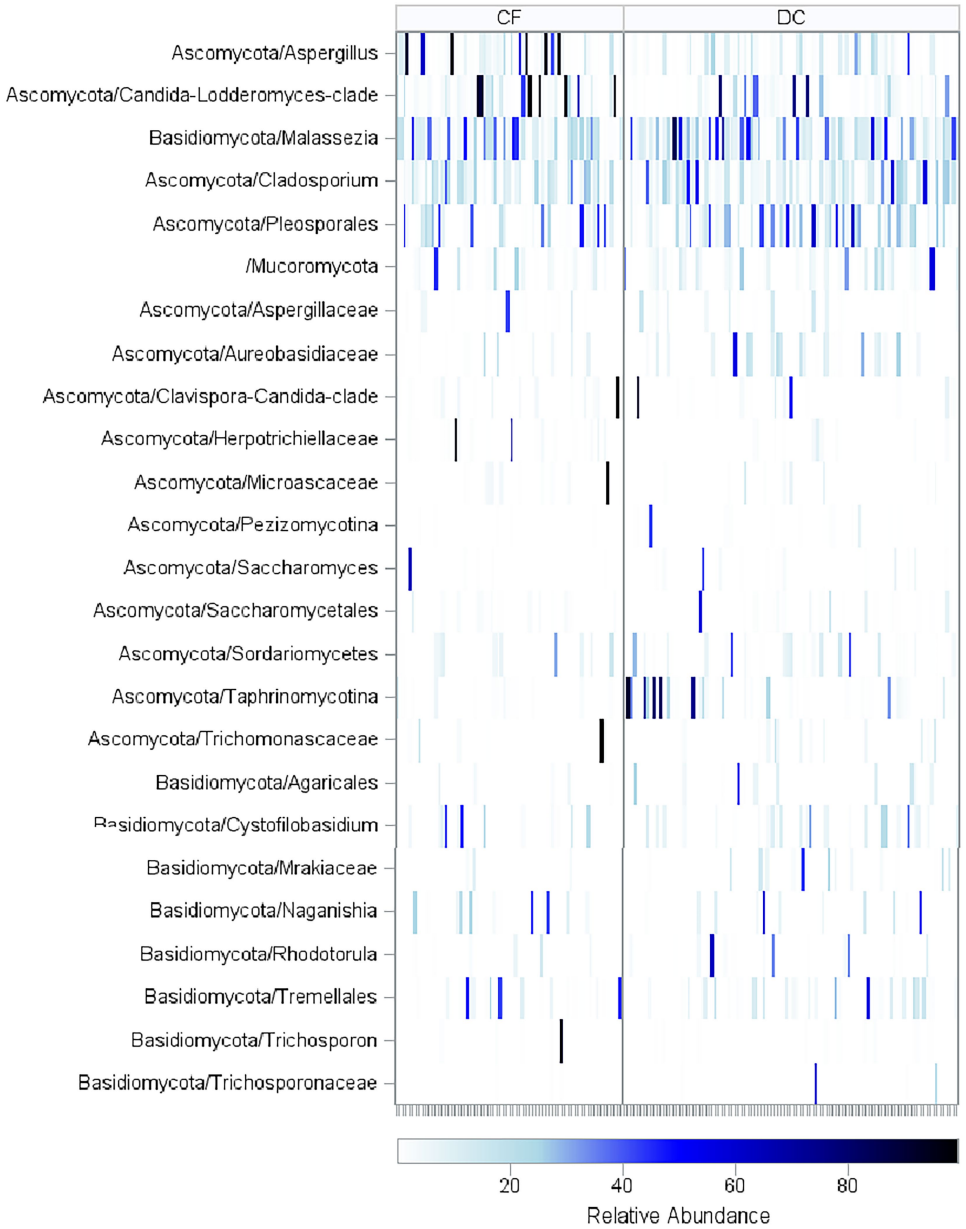
Relative abundance of fungal taxa across age. Heatmap of sequencing results for all BALF samples in CF and DC groups with samples arranged in order of increasing age (in years). Fungal Taxa that had a relative abundance of over 40% in at least one of the samples are included. PWCF had higher relative abundance of *Candida-Lodderomyces*-clade (Wilcoxon, *p* = 0.01) and *Aspergillus* (Wilcoxon, *p* = 0.06). The taxonomic OTUs reflect the resolution available from information content of the sequences and represent mutually exclusive groups, there is no overlap between sequencing counts due to placement in the taxonomic hierarchy.

The BALF mycobiome compositions as measured by SSU-rRNA are shown in Supplementary Figure 3. Including only taxa with over 1% relative abundance, the most prevalent taxa included *Malassezia,* which was found in 133 samples (82%), undifferentiated fungi not categorized into specific taxa, which was found in 119 samples (74%), *Cladosporium*, which was found in 114 samples (71%), and Pleosporales, which was found in 82 samples (51%). Relative abundances of taxa separated by CF status and organized by age are shown in Figure 3. PWCF had higher relative abundance of *Candida*-*Lodderomyces*-clade [median (IQR): 1.18 (0.17–5.08)% in PWCF compared to 0.34 (0.12–2.22)% in disease controls, Wilcoxon, *p* = 0.02] and *Aspergillus* [median (IQR): 1.26 (0.27–6.91)% in PWCF compared to 0.62 (0.13–2.44)% in disease controls, Wilcoxon, *p* = 0.07; Supplementary Figure 4].

The mycobiome compositions for the low-diversity samples, defined as those with a Shannon Diversity index under one, are shown in Supplementary Figure 5. The 13 low-diversity CF samples consisted of samples dominated by *Clavispora-Candida*-clade (*n* = 1, 8%), *Candida-Lodderomyces*-clade (*n* = 4, 31%), *Aspergillus* (*n* = 4, 31%), *Trichosporon* (*n* = 1, 8%), *Microascaceae* (*n* = 1, 8%), *Herpotrichiellaceae* (*n* = 1, 8%), and *Trichomonascaceae* (*n* = 1, 8%), while the three low-diversity DC samples consisted of samples dominated by unclassified fungi (*n* = 1, 33%), *Clavispora-Candida*-clade (*n* = 1, 33%), and *Taphrinomycotina* (*n* = 1, 33%).

### Clustering analysis

Samples with sufficient load for sequencing were clustered into hierarchical groups to evaluate potential associations between samples with similar mycobiomes and clinical variables, the annotated heatmap is shown in Supplementary Figure 6. Samples showed no clustering by CF status or other primary diagnoses. However, notable clusters included those containing high abundance of *Aspergillus* and *Candida*-*Lodderomyces*-clade, which were both associated with lower diversity (Spearman, *p* < 0.01 and *p* = 0.03) and higher TFL (Spearman, *p* = 0.11 and *p* < 0.01). Additionally, PCoA including both patient samples and background control samples showed no notable differences in the mycobiome composition between disease groups or between samples and negative controls (Supplementary Figure 7).

### Inflammation across groups and TFL

In the entire set of 191 samples, when compared to DCs, PWCF had higher white blood cells (WBC) in BALF (Wilcoxon, *p* < 0.01) and increased neutrophilic inflammation, as measured by percent neutrophils (Wilcoxon, *p* < 0.01). This was true across the range of TFL measured in samples by qPCR (Figure 2B). Percent neutrophils exhibited a slight positive correlation with TFL in DC samples (Spearman coefficient = 0.17, *p* = 0.07), but showed no correlation in PWCF (Spearman coefficient = 0.04, *p* = 0.76) which had heightened neutrophilic inflammation even at lower fungal burdens.

### Culture results

Fungal culture data were only available for 28 total BALF samples (15%) and was more frequently available in PWCF (Fisher’s exact, *p* < 0.01). When analyzed by study site, fungal culture results were more frequently available in samples from the University of Minnesota (Chi square, *p* < 0.01). When compared to bacterial culture results, fungal culture results were often accompanied by bacterial culture results (Fisher’s exact, *p* = 0.02) most of which were positive (Fisher’s exact, *p* < 0.01). Positive fungal cultures were also more frequently associated with positive bacterial cultures (Fisher’s exact, *p* = 0.046).

The 10 positive fungal cultures included *Aspergillus fumigatis* (*n* = 4, 40%), *Candida albicans* (*n* = 1, 10%), *Scedosporium apiospermum* (*n* = 1, 10%), an *Acremonium* species (*n* = 1, 10%), both *Candida guilliermondi* and *Scopulariopsis brumptii* (*n* = 1, 10%), *Candida parapsilosis* (*n* = 1, 10%), and an unspecified yeast fungus (*n* = 1, 10%). Each of the samples that were culture positive for *A. fumigatis* had DNA classified into the *Aspergillus* taxon detected in sequencing. All samples that were culture positive for *Candida* species had DNA classified into the *Candida-Lodderomyces*-clade taxon detected in sequencing. However, the sample that was culture positive for *Acremonium* species had no detectable amounts of DNA classified into the *Acremonium* taxa. Additionally, *Scedosporium* and *Scopularopsis* were not detected at all in the sequencing.

## Discussion

In this multicenter study, we demonstrated the ability to detect and identify lower airway fungal communities in PWCF and DC subjects across the pediatric age spectrum. Using a comprehensive set of BALF samples, qPCR to measure TFL, and a novel SSU-rRNA fungal sequencing technique, we measured fungal burden, detected and identified fungal communities, and observed differences in fungal load and diversity between PWCF and DC groups.

Total fungal load in the lower airways of PWCF was higher than that of DCs as has been shown previously with total bacterial load (Zemanick et al., 2017; O’Connor et al., 2021). TFL also appeared to increase with age at collection, suggesting increasing fungal burden is associated with advanced age. This is consistent with previous studies that have correlated fungal load with advanced lung disease (Amin et al., 2010; Chotirmall et al., 2010). TFL was notably higher in samples with positive fungal cultures, indicating increased fungal burden may be associated with active infection; however, high TFL values were also observed in samples with negative fungal cultures across both study populations. Similarly, both groups exhibited high prevalence of *Malassezia*, *Cladosporium*, Pleosporales, and undifferentiated fungi. While the undifferentiated fungi could be further classified in future studies, *Malassezia* is associated with a variety of human skin conditions, including dandruff (Gupta et al., 2004), *Cladosporium* is found in both indoor and outdoor environments and known to be associated with decaying plants and spoiled food (Bullerman, 2003; Prasannath et al., 2021), and Pleosporales is responsible for black mold commonly found in indoor environments. Additionally, ordination analysis with background reagents revealed little difference between background and patient sample communities. None of the reagent controls exhibited amplification during PCR, which complicates interpretation of this negative signal. However, the increase in TFL with age, the TFL present in negative cultures, the taxa associated with environmental sources, and the inability of ordination analysis to separate samples from background suggest the potential accumulation of fungal DNA inhaled from the environment in the lower airways. It is unclear if the fungal load is representative of active infection, harmless fungi, or ambient DNA accumulated from the environment. A potential hypothesis is an ongoing fungal bioaccumulation process in the lungs resulting in the presence of a shared lower airway fungal background. The increased fungal burden in CF could represent a difference in CF lower airway physiology, whereby PWCF inhale fungal DNA or living fungi from the environment, and, due to the impaired mucociliary clearance in the CF lung, fungi, and fungal DNA remain in the lung and accumulate over time. This is consistent with previous findings in adult CF sputum that proposed inhalation as a driving force of the CF airway mycobiome composition (Kramer et al., 2015).

While PWCF had increased fungal burden, in both groups, SSU-rRNA sequencing revealed complex and diverse airway mycobiomes with similar Shannon diversities. A subset of PWCF showed decreases in diversity with age, as we previously showed with bacterial sequencing (O’Connor et al., 2021). This decreased diversity at older ages was associated with the abundance of *Candida* and *Aspergillus* which appear to dominate the CF airway. While PWCF exhibited increased abundance of *Candida* and *Aspergillus,* which is consistent with previous culture and sputum sequence-based studies (Willger et al., 2014; Al Shakirchi et al., 2020; Breuer et al., 2020; Cuthbertson et al., 2021), overall samples did not cluster based on CF status, indicating PWCF and DCs did not have notably different airway mycobiomes. In PWCF and DCs, while there were similarities in the diverse mycobiomes composed of potential environmental sources, our data demonstrated different host responses, as measured by neutrophilic inflammation. Samples from PWCF were associated with increased neutrophilic inflammation, as we previously reported, across varying TFL and Shannon diversity measures (O’Connor et al., 2021). While neutrophilic inflammation was positively correlated with TFL in DCs, in PWCF, high neutrophilic inflammation was observed even at low TFL values suggesting heightened neutrophilic inflammation independent of fungal burden. Those findings are also consistent with other human and animal studies which show that the CF airway exhibits heightened inflammation even at times of clinical stability and no active infection (Sagel and Accurso, 2002; Pillarisetti et al., 2011; Rosen et al., 2018).

It is worth noting that most BALF samples did not have fungal culture data available, which could be because the samples were collected before fungal culture was prevalent in clinical care. Inconsistencies in the use of fungal culture by CF status and institution further underscore the importance of establishing a standardized approach to detect airway fungi (Hong, 2022).

Our study is not without limitations. Given the nature of a multicenter study, BALF sample collection was not standardized, which may have introduced variability given the different lobe(s) of lung that were sampled. Research-based bronchoscopy is not possible in the United States in children, making identifying a large set of BALF samples available for study in children with and without CF difficult. Our reliance on a convenience sample set of banked BALF collected as part of previous clinical research studies limits the availability of complete and comprehensive associated clinical data. This directly informs inconsistencies in documentation of diagnoses, culture results, and completeness of data that may have impacted our findings. Also, all BALF samples were collected from clinically indicated bronchoscopies obtained most often during times of illness, meaning fungal communities identified may not represent a baseline state of health. Similarly, because DC samples were collected from bronchoscopies performed for a variety of indications, taxa detected do not represent a normal airway mycobiome and could be impacted by the heterogeneity of the underlying diagnoses and indications. Also, our study did not account for the complex relationship between airway fungi and bacteria, which can greatly impact lower airway infection and inflammation. Previous work has highlighted the interplay between *Aspergillus* and traditional CF pathogens (Amin et al., 2010; Soret et al., 2020). The interactions between airway bacterial and fungal communities could also explain the differences observed in host response. Additional multi-omic correlations between the mycobiome and the microbiome will be explored as next steps. Lastly, while SSU-rRNA sequencing provided us with the ability to detect multiple fungal communities present in the lower airways, it is still unclear if the DNA detected represents active infection, harmless fungi, or DNA accumulated from the environment. The DNAs utilized in this study were previously generated to examine bacterial communities and thus the extraction approach was not tailored for disruption of fungal cell structures. This may influence the community structure observed due to inefficient lysis.

In this study, we used a novel fungal SSU-rRNA sequencing approach to characterize the lower airway mycobiomes in BALF samples from pediatric PWCF and DC subjects. While questions regarding fungi and its role in CF airway infection and inflammation remain, our study explored the use of molecular sequencing-based approaches to elucidate the relationship between fungi and CF pathophysiology and will inform future study.

## Data availability statement

The data presented in the study are deposited in the NCBI Sequence Read Archive, accession number PRJNA922524 (https://www.ncbi.nlm.nih.gov/bioproject/PRJNA922524).

## Ethics statement

The studies involving human participants were reviewed and approved by Children’s Hospital Colorado Institutional Review Board and the University of Minnesota Institutional Review Board. Written informed consent to participate in this study was provided by the participants’ legal guardian/next of kin.

## Author contributions

TL, JH, DF, and BW contributed to conception and design of the study. JH and DF developed 18S and qPCR pipelines and generated the data. JO’C managed the sample database and sample transfers between institutions. BW and JO’C performed the statistical analyses. JO’C wrote the first draft of the manuscript. BW and JH wrote the sections of the manuscript. All authors contributed to the article and approved the submitted version.

## Funding

The authors acknowledge funding provided by the Cystic Fibrosis Foundation (CFF LAGUNA17A0) and National Institutes of Health (NIH R01HL136499).

## Conflict of interest

The authors declare that the research was conducted in the absence of any commercial or financial relationships that could be construed as a potential conflict of interest.

## Publisher’s note

All claims expressed in this article are solely those of the authors and do not necessarily represent those of their affiliated organizations, or those of the publisher, the editors and the reviewers. Any product that may be evaluated in this article, or claim that may be made by its manufacturer, is not guaranteed or endorsed by the publisher.

## Footnotes

^1^ http://support.illumina.com/sequencing/sequencing_software/igenome.ilmn
