# Supplementary material for: Detection and identification of fungi in the lower airway of children with and without cystic fibrosis
Source: Front Microbiol. 2023 Feb 9;14:1119703. doi: 10.3389/fmicb.2023.1119703 (PMC9948248; doi:10.3389/fmicb.2023.1119703)
Supplement: Supplementary file 1 [file Data_Sheet_1.docx]

# **Supplementary Material:**


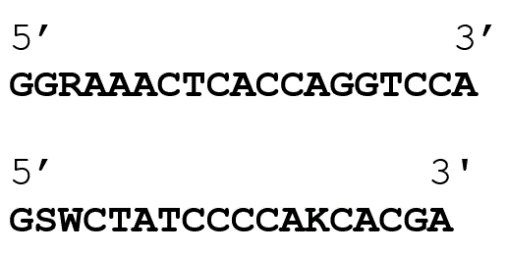


**Supplementary Figure 1:** Primer sequences for SSU-rRNA gene sequencing

|  | **DCs (n = 101)** | **CF (n = 60)** | **P-value** |
| --- | --- | --- | --- |
| **Age years, median (range)** | **6.0 (0.1-21.0)** | **9.8 (0.9-19.7)** | **<0.001** |
| **<2 years, number (%)** | **24 (24%)** | **3 (5%)** | **<0.001*** |
| **2-5 years, number (%)** | **26 (26%)** | **9 (15%)** |  |
| **6-10 years, number (%)** | **29 (29%)** | **23 (38%)** |  |
| **11-17 years, number (%)** | **13 (13%)** | **20 (33%)** |  |
| **18 years and older, number (%)** | **9 (9%)** | **5 (8%)** |  |
| **Female, number (%)** | **48 (48%)** | **39 (65%)** | **0.032** |
| **Weight (kg), median (range) (data available)** | **19.2 (4.4-70.9) (N=101)** | **28.9 (13.5-74.7) (N=46)** | **<0.001** |
| **Height (cm), median (range) (data available)** | **111.0 (55.0-181.0) (N=96)** | **134.0 (97.8-175.4) (N=46)** | **<0.001** |
| **Genotype, data available** | **N/A** | **N=58** | **N/A** |
| **F508del/F508del, number (%)** | **N/A** | **34 (59%)** | **N/A** |
| **F508del/other, number (%)** | **N/A** | **21 (36%)** | **N/A** |
| **Other/other, number (%)** | **N/A** | **3 (5%)** | **N/A** |
| **FEV1 % predicted, median (range) (data available)** | **77.5 (43.0-109.0) (N=22)** | **86.0 (35.0-131.0) (N=47)** | **0.322** |
| **BALF Cell Counts, data available** | **N=99** | **N=51** | **N/A** |
| **White blood cells, median (range) (data available)** | **230.0 (6.0-8122.0) (N=99)** | **1250.0 (66.0-51500.0) (N=51)** | **<0.001** |
| **Percent Neutrophils, median (range) (data available)** | **9.5 (1.0-99.0) (N=88)** | **79.0 (3.0-99.0) (N=49)** | **<0.001** |
| **Percent Lymphocytes, median (range) (data available)** | **10.0 (1.0-66.0) (N=94)** | **3.5 (0-28.0) (N=38)** | **<0.001** |
| **Positive Bacterial Culture, number (% of those with data available) (number with data available)** | **34 (34%) (N=100)** | **26 (47%) (N=55)** | **0.105** |
| **Positive Fungal Culture, number (% of those with data available) (number with data available)** | **4 (80%) (N=5)** | **6 (29%) (N=21)** | **0.055*** |
| **Antibiotic Use number (% of those with data available) (number with data available)** | **28 (88%) (N=32)** | **28 (47%) (N=59)** | **<0.001*** |

**Supplementary Table 1: Samples with Fungal Sequencing:** Data are presented as n, median (range) or n (%), unless otherwise stated. CF: cystic fibrosis; FEV1: forced expiratory volume in 1 s; BALF: bronchoalveolar lavage fluid; * P-value calculated using Fisher’s exact test


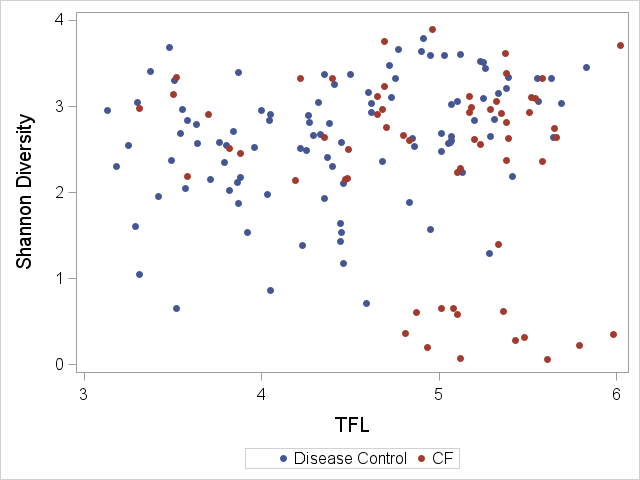


**Supplementary Figure 2: Load and Diversity.** Shannon Diversity vs. TFL in PWCF and DC samples


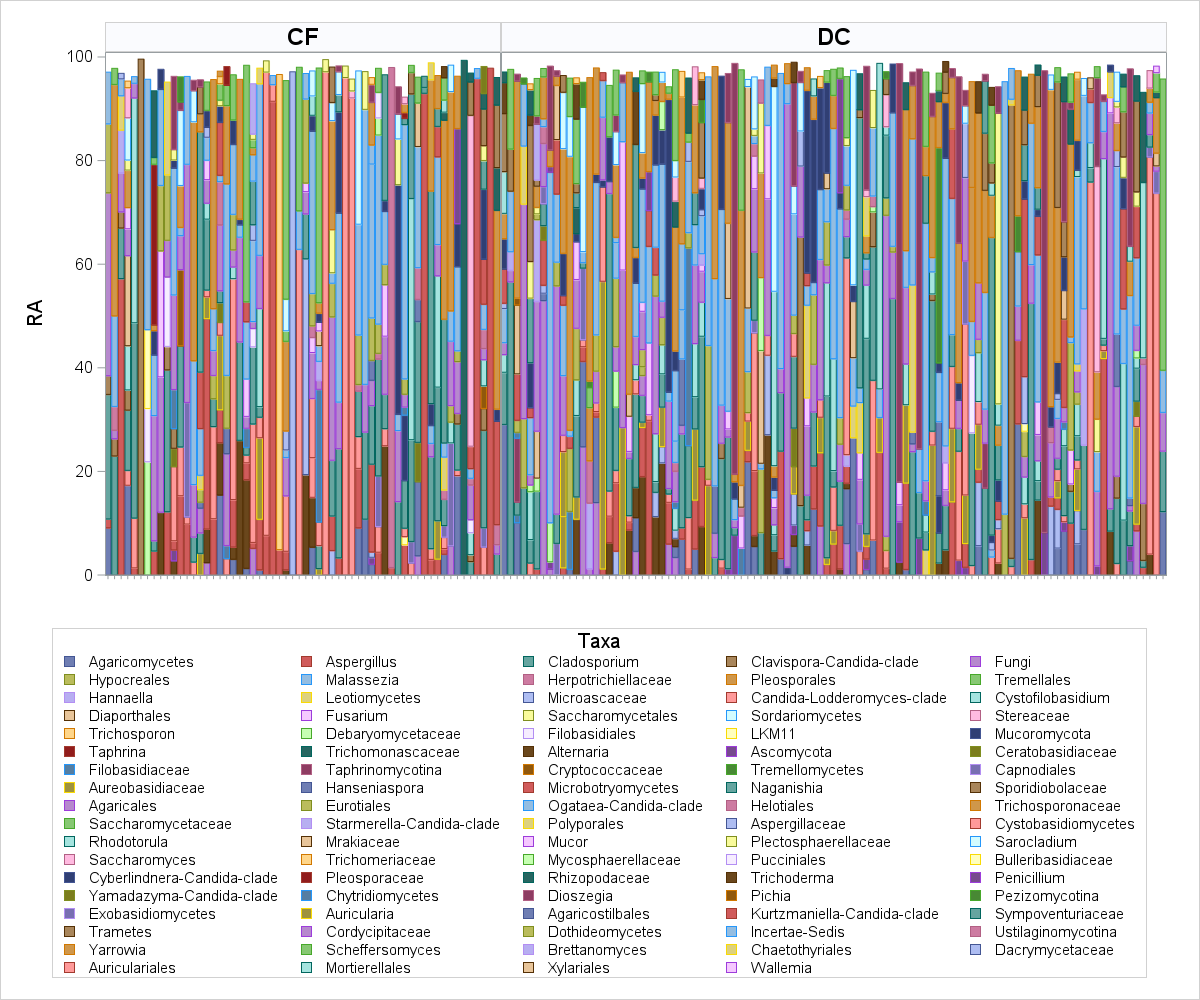


**Supplementary Figure 3: Mycobiome Composition.** SSU-rRNA sequencing results, including relative abundances of taxa in CF and DC subjects organized by age, only taxa with over 1% RA were included for simplicity


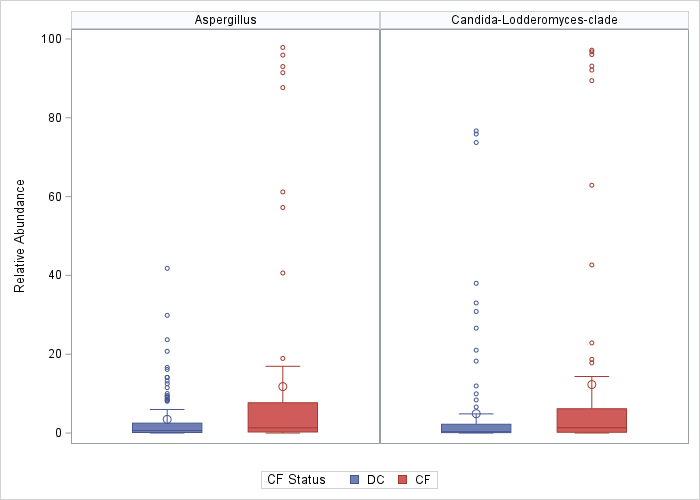


**Supplementary Figure 4: Relative Abundances of *Aspergillus* and *Candida*.** Box plots of relative abundance of *Aspergillus* and *Candida*, two common CF pathogens, grouped by CF status


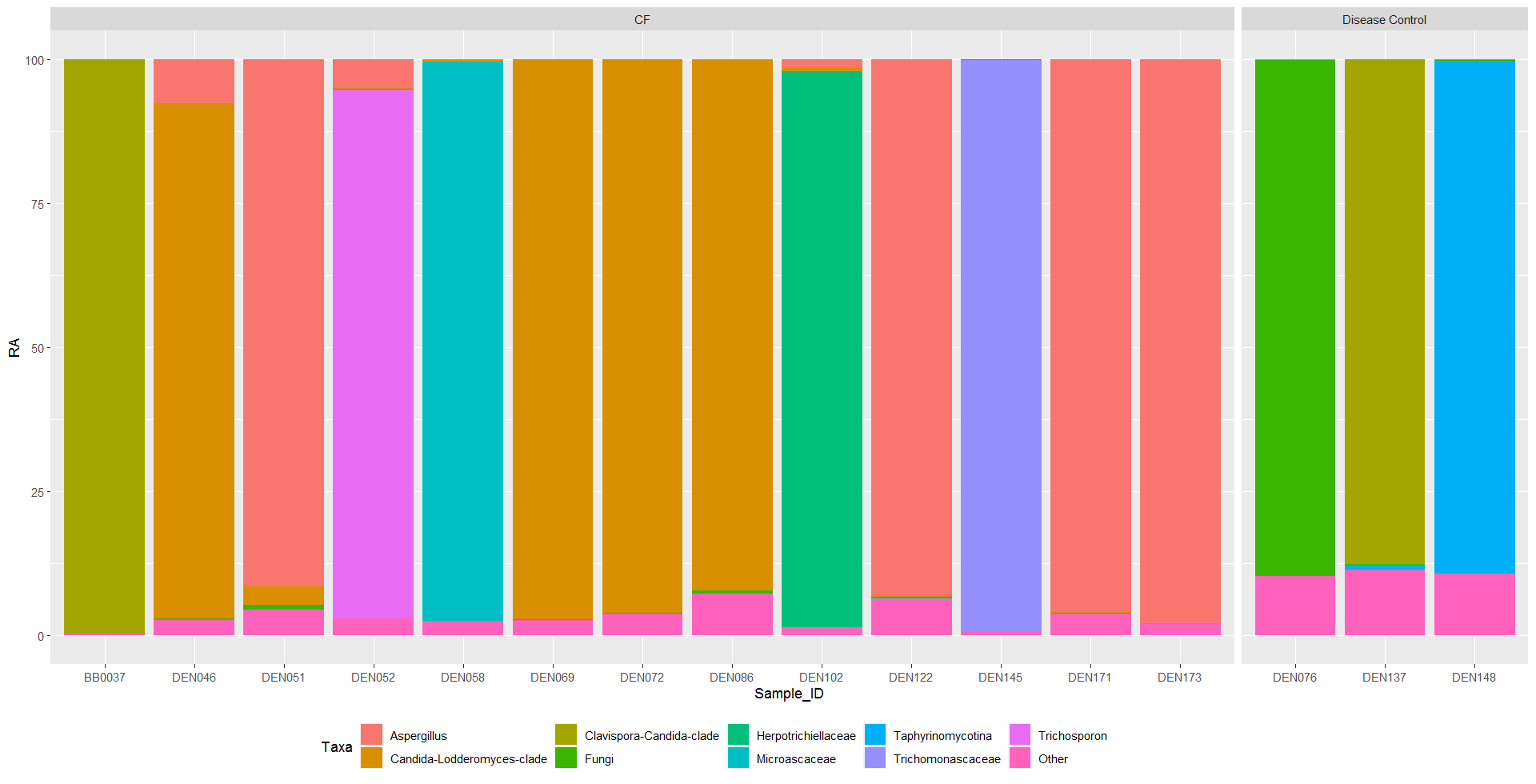


**Supplementary Figure 5: Mycobiome Composition of Low-Diversity Samples.** SSU-rRNA sequencing results, including relative abundances of taxa in CF and DC subjects, only dominate taxa that were over 50% RA in at least one sample were included, while the remaining taxa were categorized as “Other” for simplicity


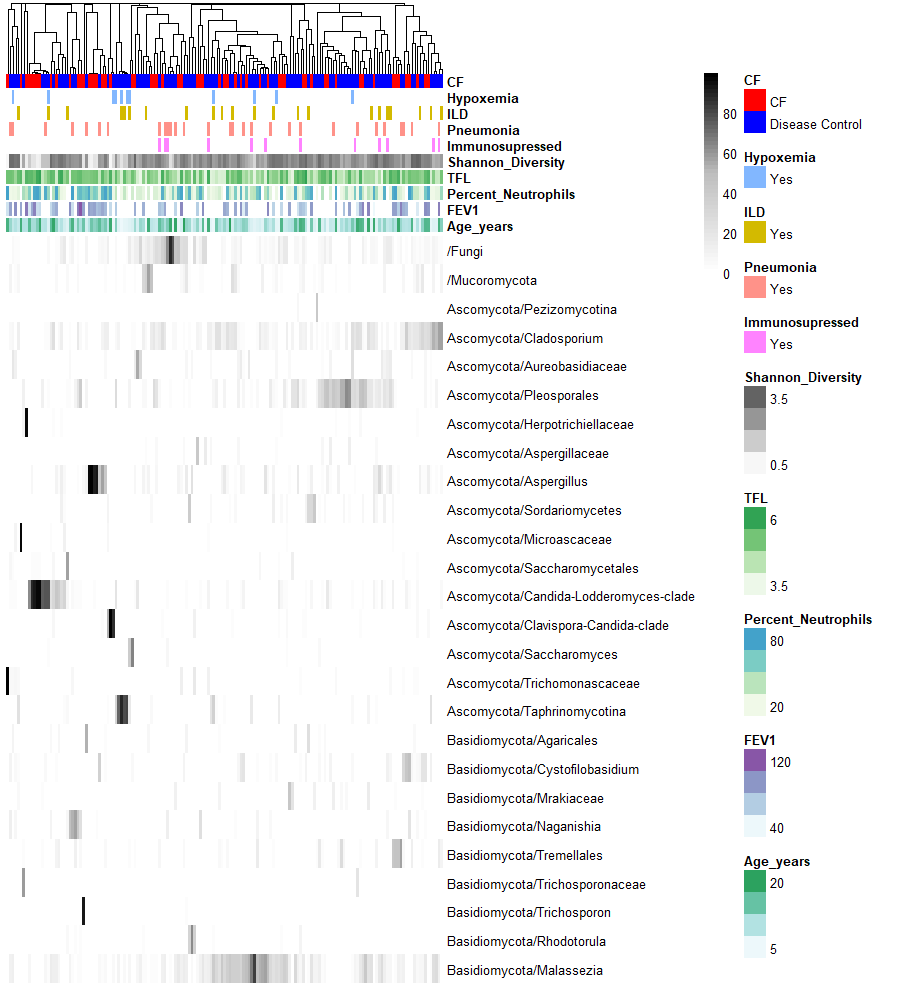


**Supplementary Figure 6: Unsupervised Clustering Analysis.** Subjects with similar mycobiome groups were clustered together based on Morisita-Horn Beta Diversity as indicated in the dendrogram at the top of the figure. Subjects closest together in the lowest branches of the tree have similar mycobiomes, the heatmap shows the relative abundance for selected fungi (RA > 40% in at least one sample) for each subject. The heatmap is annotated with TFL, Shannon Diversity, demographic information, and clinical data, none of which were included in the unsupervised clustering. There are no discernable associations between subjects with similar mycobiomes and clinical factors. Abbreviations: ILD- Interstitial lung disease; TFL – total fungal load; FEV – forced expiratory volume


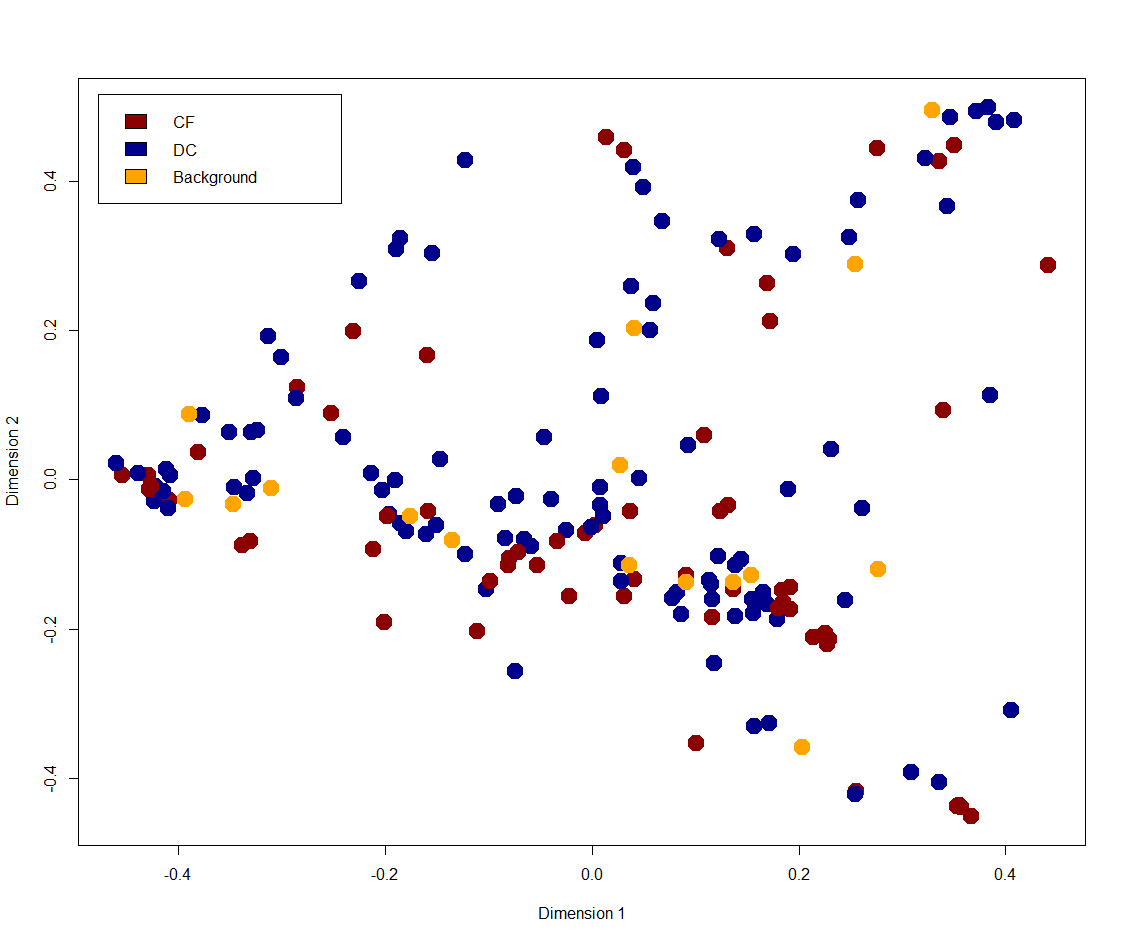


**Supplementary Figure 7: Ordination Analysis.** Clustering analysis of samples based on SSU-rRNA sequencing using the Morisita-Horn Beta Diversity Measure including CF samples, DC samples, and negative controls.
